# Multi-categorical deep learning neural network to classify retinal images: A pilot study employing small database

**DOI:** 10.1371/journal.pone.0187336

**Published:** 2017-11-02

**Authors:** Joon Yul Choi, Tae Keun Yoo, Jeong Gi Seo, Jiyong Kwak, Terry Taewoong Um, Tyler Hyungtaek Rim

**Affiliations:** 1 Department of Electrical and Computer Engineering, Seoul National University, Seoul, South Korea; 2 Institute of Vision Research, Department of Ophthalmology, Yonsei University College of Medicine, Seoul, South Korea; 3 Department of Electrical & Computer Engineering, University of Waterloo, Waterloo, Ontario, Canada; Harbin Institute of Technology Shenzhen Graduate School, CHINA

## Abstract

Deep learning emerges as a powerful tool for analyzing medical images. Retinal disease detection by using computer-aided diagnosis from fundus image has emerged as a new method. We applied deep learning convolutional neural network by using MatConvNet for an automated detection of multiple retinal diseases with fundus photographs involved in STructured Analysis of the REtina (STARE) database. Dataset was built by expanding data on 10 categories, including normal retina and nine retinal diseases. The optimal outcomes were acquired by using a random forest transfer learning based on VGG-19 architecture. The classification results depended greatly on the number of categories. As the number of categories increased, the performance of deep learning models was diminished. When all 10 categories were included, we obtained results with an accuracy of 30.5%, relative classifier information (RCI) of 0.052, and Cohen’s kappa of 0.224. Considering three integrated normal, background diabetic retinopathy, and dry age-related macular degeneration, the multi-categorical classifier showed accuracy of 72.8%, 0.283 RCI, and 0.577 kappa. In addition, several ensemble classifiers enhanced the multi-categorical classification performance. The transfer learning incorporated with ensemble classifier of clustering and voting approach presented the best performance with accuracy of 36.7%, 0.053 RCI, and 0.225 kappa in the 10 retinal diseases classification problem. First, due to the small size of datasets, the deep learning techniques in this study were ineffective to be applied in clinics where numerous patients suffering from various types of retinal disorders visit for diagnosis and treatment. Second, we found that the transfer learning incorporated with ensemble classifiers can improve the classification performance in order to detect multi-categorical retinal diseases. Further studies should confirm the effectiveness of algorithms with large datasets obtained from hospitals.

## Introduction

Retina is a photosensitive layer of optic nerve tissue lining in the inner surface of the eyeball. Retinal damages due to various diseases can eventually lead to irreversible vision loss. As population aging has emerged as a major demographic trend worldwide, patients suffering from chorioretinal diseases such as age-related macular degeneration (AMD) and diabetic retinopathy (DMR) are expected to increase in the future [1]. AMD is can cause blindness [2]. DMR, which a common lifestyle disease, is also the a major cause of blindness in patients with diabetes mellitus [3]. Other retinal diseases including retinal vessel occlusion, hypertensive retinopathy, and retinitis are significant causes of vision impairment. If early diagnosis and treatment are implemented prior to the initial stage of blindness progression, visual loss can be avoided in many cases. Hence, more precise screening program is required for early treatment in high-risk group in an effort to reduce socioeconomic burdens of visual loss caused by retinal diseases. DMR screening that uses fundus photograph is universally adopted for diabetes patients. Moreover, screening such as AMD is the most appropriate approach for early intervention in the asymptomatic stage [4]. Conducting AMD screening and DMR screening is cost-effective in a public health setting [5]. However, manual analysis for multiple fundus photographs for an accurate screening requires a great deal of efforts of ophthalmologists.

Many previous studies have focused on automated detection of retinal diseases by using machine learning algorithms in order to analyze a large number of fundus photographs taken from retinal screening programs [6,7]. Various machine learning algorithms—K-nearest neighbor algorithm, Naive Bayes classifier, artificial neural network (ANN), and support vector machine (SVM)—were applied to automated retinal disease detection [8]. However, only a few studies developed machine learning models for AMD detection; whereas, most studies devoted in identifying DMR [9].

Deep learning for analyzing medical images appeared in the field of machine learning technique [10]. There were several reports on introduced ANN models to mark the difference between glaucoma and non-glaucoma [11,12]. A glaucoma research group reported visual field analysis by using deep feed-forward neural network to discover preperimetric glaucoma [13]. An automated deep convolutional neural network (CNN) was applied in the grading severity of nuclear cataract [14]. Moreover, a similar technique model identifying retinopathy of prematurity by using babies’ retinal images was developed [15]. According to the recent outcome from Abramoff’s research team, this learning technique demonstrated better performance in terms of automated DMR detection than previous algorithms [16]. The Google research team has introduced the advanced deep learning model capable of diagnosing DMR as well as human ophthalmologists [17]. Using similar deep learning techniques, fundus photographs and optical coherence tomography were used for analyzing the AMD [18,19]. Yet all the studies for retinal image classification selected binary classification through which “one disease versus normal” problems were settled. Although studies performed in the past released the outcome that high performances of classification in controlled experimental settings, it is practically difficult to apply the binary classification model into the real clinical setting where visiting patients suffer from various retinal diseases. Nonetheless, studies about multi-categorical classification aiming at identifying ocular diseases have been very limited.

In this study, we applied deep learning using a state-of-the-art CNN for fundus photography analysis in multi-categorical disease settings. This paper articulates a pilot study designed for deep learning assessment on multi-categorical classification by using small open retinal image database.

## Methods

**Fig 1:**
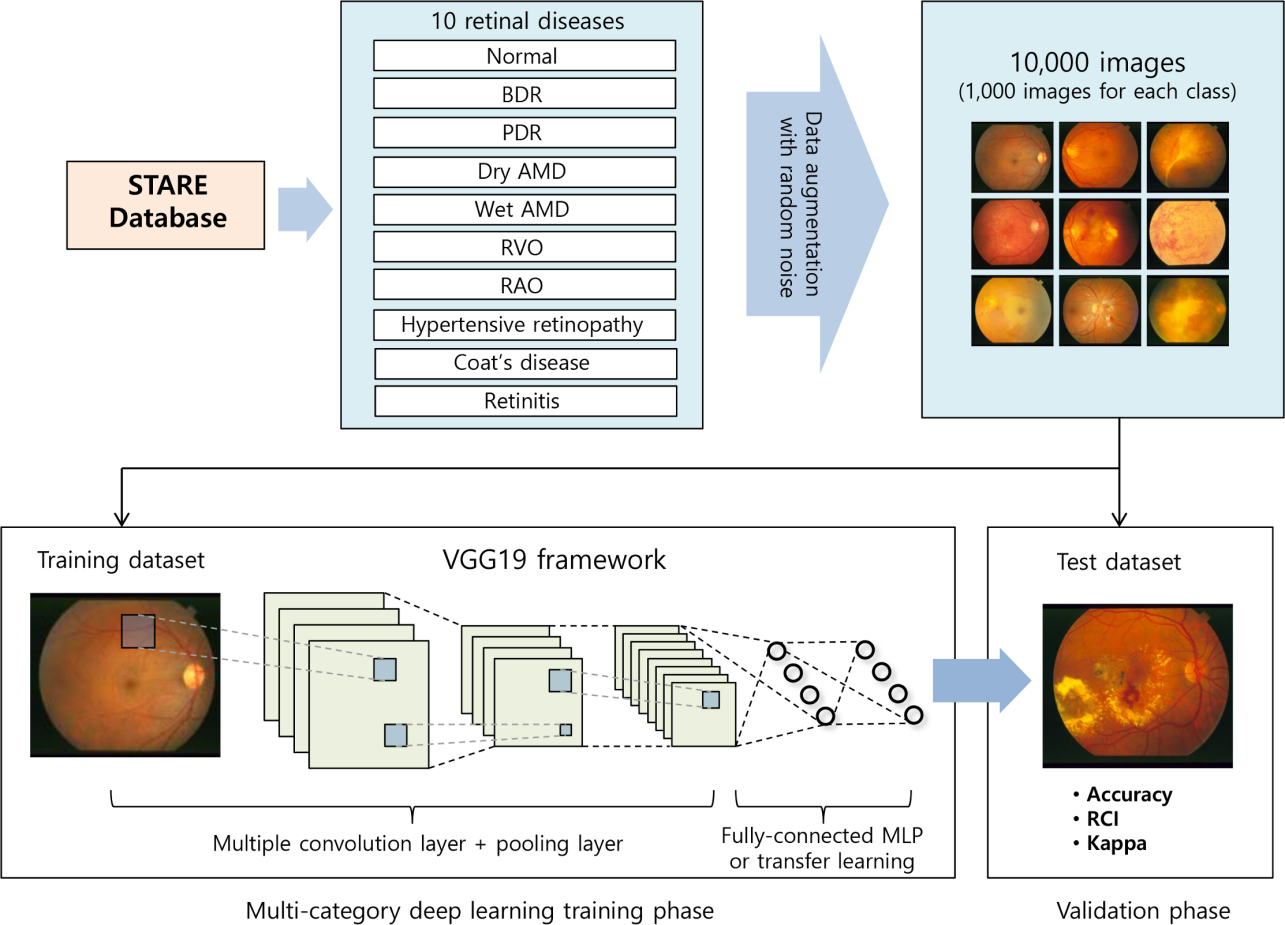
Illustration of the proposed procedure in this study.

We utilized publically available retinal image database at STructured Analysis of the REtina (STARE) project (available at http://www.ces.clemson.edu/~ahoover/stare) [20] in order to evaluate a multi-categorical deep learning model. Fig 1 shows a flow diagram of the proposed system. The experimental process complied with the Declaration of Helsinki. The Ethics committee approval was not required, because researchers instead used public database. The STARE project aimed to develop an image-understanding system to distinguish retinal diseases from fundus images. Database is comprised of retinal color images acquired by a TRV-50 fundus camera (Topcon Corp., Tokyo, Japan) at a 35 degrees field with a resolution of 605 x 700 pixels. The database contains 397 images in 14 disease categories including emboli, branch retinal artery occlusion (BRAO), cilio-retinal artery occlusion, branch retinal vein occlusion (BRVO), central retinal vein occlusion (CRVO), hemi-CRVO, background diabetic retinopathy (BDR), proliferative diabetic retinopathy (PDR), arteriosclerotic retinopathy, hypertensive retinopathy, Coat’s disease, macroaneurism, choroidal neo-vascularization(CNV), and the other retinal status.

However, there are several problems on original categorization. First, categorization was unequally distributed. For example, dataset includes only a single image of cilio-retinal artery occlusion, while more than 60 images were included into the BDR. Second, important disease groups, such as dry AMD (drusens in macula), wet AMD, central retinal arterial occlusion (CRAO), and retinitis were not classified. Therefore, two ophthalmologists (T.K.Y and J.G.S) reviewed all images and assigned new categories. Several categories were removed due to extremely small number of images such as emboli without vessel occlusion (6 images), cilio-retinal artery occlusion (1 image), arteriosclerotic retinopathy without DMR (6 images) and macroaneurism (8 images). BRVO, CRVO, and hemi-CRVO categories were incorporated into a single category of retinal vein occlusion (RVO) and categories of BRAO. Furthermore, CRAO was also integrated into retinal arterial occlusion (RAO). We excluded 28 low-resolution images and 69 the ambiguity of retinal images that present overlapped multiple diseases. At the last stage, we involved remaining 279 images and classified them into 10 categories including normal (25 images), BDR (63 images), PDR (17 images), dry AMD (25 images), wet AMD (48 images), RVO (38 images), RAO (12 images), hypertensive retinopathy (19 images), Coat’s disease (12 images) and retinitis (20 images).

Researches who noted that deep learning in medical image analysis used trained deep CNN models from scratch. The recent development of rapid parallel solvers with GPU promoted to train huge parameters in deeper CNN models. State-of-the-art deep learning algorithms presented in the ImageNet Large Scale Visual Recognition Competition (ILSVRC) originally centered on multi-categorical (or multiclass) classification problems since data provided by the ILSVRC contains more than a million training images from 1,000 object categories [21]. We used the deep learning CNN model with 19 layers (VGG-19) along with MatConvNet (available at http://www.vlfeat.org/matconvnet). Oxford University Visual Geometry Group developed MatConvNet, an open toolbox that implements new CNN including VGG-16, and VGG-19, placing second in the ILSVRC in 2014. VGG-19 has been widely adopted to solve image classification problems as the GoogLeNet, which won ILSVRC prize in 2014. There was no significant difference in terms of performance between VGG-19 and GoogLeNet. Moreover, VGG-19 is simple and efficient for individual user [22]. VGG-19 uses 224 x 224 pixels with RGB 3-channel images as input variables. MatConvNet also provides AlexNet, a standard CNN architecture as well as a winner of ILSVRC in 2012. There are three types of layers of all CNN models in this study: convolutional (computing the output of the connected local input neurons reading patterns), max pooling (sub-sampling the inputs) and fully connected layers (allocating final scores of each class). VGG-19 is composed of 16 convolutional layers and 3 fully connected layers.

In this study, we compared and analyzed four distinct deep learning models. VGG-19 and AlexNet were operated based on a stochastic gradient descent (SGD) method. Other models adopted a transfer learning technique based on a pre-trained VGG-19 model. If the models were trained ones by using SGD, we adopted a pre-trained model as a starting point for learning the network weights. The pre-trained model, previously trained on a subset of current ImageNet database (provided by MatConvNet), was further processed with fundus image dataset. VGG-19 and AlexNet were algorithms applied in training by using momentum 0.9, and a fixed learning rate of 10^−6^ for 50 epochs.

A machine learning model can apply accumulated knowledge to a new task domain by applying transfer learning technique [23]. We retain original 279 retinal images from 10 categories, which are relatively small to deal with a CNN with millions of parameters. Thus, they should be adjusted optimally. A transfer learning by using pre-trained CNN can contribute to avoid the problem associated with a few dataset in medicine. Previous studies suggested that CNN intermediate layer outputs can function as input features to train other classifiers and that this skill demonstrated satisfactory accomplishment performance in dealing with various problems; thus, we used a pre-trained VGG-19 model [23,24]. Although this pre-trained model was optimally programmed to identify 1000 objects in ImageNet, our hypothesis is that several pre-trained texture features might be apt for analysis on photographs. When the CNN served as a feature extractor, multiclass random forest (RF) and SVM models were trained by utilizing the 4096 input features from the last covered layer of pre-trained VGG-19 model. SVM is a universally well-established technique based on mapping data in a higher dimensional space via a kernel function and selecting the maximum-margin hyper-plane dividing training data. Multiclass SVM adopted one-vs-one design that builds up binary SVM models for all pairs of classes. A decision function of one-vs-one design assigns an instance to a class that involves countless votes [25]. RF refers to a robust and powerful multiclass classification method that promotes many classification trees from random subsets of predictors and bootstrap samples. Previous studies revealed that these two multiclass classifier are the most robust techniques that outperforms other algorithms including decision trees, k-nearest neighbor, and back-propagation neural networks [26].

We applied ensemble classifiers to the transfer learning process to enhance the performance. Ensemble methods have been proved to be a potent tool to stabilize and improve the performance of machine learning classifiers [27]. By using the disease-labeled image data and 4096 input features from the last covered layer of pre-trained VGG-19 model, we trained ensemble SVM classifiers by complying with clustering and voting approaches (iRSpot-EL) [28], K-means clustering with dynamic selection strategy (D3C) [29], multiple kernel learning [30], and AdaBoost (deep SVM) [31]. We replaced the previous ensemble classifier with preserving the structure of iRSpot-EL (modified iRSpot-EL). Previous researchers defined the distance between two classifiers *C*(*i*) and *C*(*j*) as follows:

$$Distance(C(i),C(j))=1-\frac{1}{2m}\sum_{k=1}^{m}\left(d_{ik}\Delta d_{jk}\right)$$ (1)

where *m* represents the number of training samples, *d*_*ik*_ refers to the misclassification probability of classifier *C*(*i*) on the *k*th sample, and *d*_*ik*_Δ*d*_*jk*_ can be calculated as follows:

$$d_{ik}\Delta d_{jk}=\left\{ \begin{array}{c} d_{ik}+d_{jk},\;if\;C(i)\;and\;C(j)\;incorrectly\;predicts\;the\;kth\;sample\\ 0,\;otherwise \end{array}\right.$$ (2)

Based on the distance, the affinity propagation clustering algorithm was estimated. 300 different multiclass SVM models were constructed by using following parameter combinations in order to set up different multi-categorical classifiers:

$$\left\{\begin{array}{c} OVO\\ OVA\\ DAG \end{array}\right\}\;RBF\_SVM\;using\;\left\{ \begin{array}{c} -5<\log\left(C\right)\leq 5\;with\;step\;\Delta =1\\ -5<\log\left(\sigma \right)\leq 5\;with\;step\;\Delta =1 \end{array}\right.$$ (3)

where *OVO* represents one-versus-one classifier, *OVA* stands for one-versus-all classifier [32], *DAG* notes directed acyclic graph classifier [33], and *RBF_SVM* refers to radial basis function SVM with a penalty parameter *C* and scaling factor *σ*. After 300 different multiclass SVM models were acquired, they were classified into seven clusters in compliance with affinity propagation clustering [34]. The ensemble process was implemented via the following fractional votes:

$$Y=\frac{1}{7}\sum_{i=1}^{7}F_{i}P_{i}$$ (4)

where *P*_*i*_ represents the probability from the classifier *C*(*i*), and *F*_*i*_ refers to its fraction used, which was optimized on the validation sets. This ensemble multi-categorical classifier is compared to single SVM, RF, and the above ensemble methods. We also applied feature selection methods with subsets of the top-ranked 1024, 2048, 3072, and 4096 input features in order to examine how feature dimensionality and change the performance. Features were selected by using Kruskal-Walis one-way ANOVA (KW), ratio of features between-categories to within-category sum of squares (BW) [35], and Max-Relevance-Max-Distance (MRMD) [36]. All parameters of each ensemble method were highly tuned to promote the performance.

We augmented data by oversampling images with translation, rotation, brightness change, and additive Gaussian noise due to reduced size of fundus image dataset for training CNN models [37]. Data augmentation is a widely used approach to boost the generalization of deep learning models. We randomly retrieved transformed 1000 fundus images per each disease class because of the imbalance of data problems. Specifically, we obtained samples with translation from the range [-10%, +10%] of the image width, with rotation from [-15°, +15°], and with brightness change from a range of [-10%, +10%]. Additive Gaussian noise has a uniformly sampled sigma from [0, 0.04]. All images were organized according to the input size of the pre-trained model (224 x 224 pixels) in the course of oversampling.

The measurement of multi-categorical problems was based on the accuracy, relative classifier information (RCI), and Cohen's kappa metric [38]. Accuracy is a standard metric for evaluation of a classifier. It is defined as follows:

$$Accuracy=\frac{\sum_{i}q_{ii}}{\sum_{ij}q_{ij}}$$ (5)

where the element *q*_*ij*_ refers to the number of test times and test input actually labeled *C*_*i*_ is *C*_*j*_ noted by the classifier, and these elements organize the confusion matrix. Although it is easy to notice the accuracy, it cannot give full accounts on the actual performance in multi-categorical problems. The RCI is an entropy-based measure applicable to multi-categorical decision problems [39]. This quantifies how much uncertainty of classification had been reduced by a machine learning classifier [25]. It is defined as follows:

$$RCI=\sum_{i}-\frac{\sum_{j}q_{ij}}{\sum_{ij}q_{ij}}\log\left(\frac{\sum_{j}q_{ij}}{\sum_{ij}q_{ij}}\right)-\sum_{j}\left(\frac{\sum_{i}q_{ij}}{\sum_{ij}q_{ij}}\times \sum_{i}-\frac{q_{ij}}{\sum_{i}q_{ij}}log(\frac{q_{ij}}{\sum_{i}q_{ij}})\right)$$ (6)

where *log* refers to natural logarithm transformation. RCI represents the performance with unbalanced classes capable of distinguishing among different misclassification distributions. Cohen’s kappa is an alternative to classification rate that compensates for random hits [40]. It is defined as follows:

$$Kappa=\frac{\sum_{ij}q_{ij}\times \sum_{i}q_{ii}-\sum_{ij}\left(\sum_{i}q_{ij}\times \sum_{j}q_{ij}\right)}{{\left(\sum_{ij}q_{ij}\right)}^{2}-\sum_{ij}\left(\sum_{i}q_{ij}\times \sum_{j}q_{ij}\right)}$$ (7)

Kappa is a standard meter for a multi-categorical problem generally applied in several fields such as brain-computer interface.

Matlab 2016a (Mathworks, Natick, MA, USA) was prepared to perform the algorithms. When we trained binary classifiers, we maximized the Youden's index to select cut-off points and granted equivalent portions to sensitivity and specificity [41]. We used MedCalc 12.3 (MedCalc, Mariakerke, Belgium) for Receiver Operating Characteristic (ROC) analysis. When we conducted ROC analysis, we divided all dataset (10,000 images) into training dataset (70%) and test dataset (30%). When training process was completed, test dataset was valid to create ROC curves. This process demonstrated that our training process did not derail an ordinary deep learning study process if we obtained the similar classification performance like the previous binary classification research.

We compared four deep learning models: transfer learning with random forest based on VGG-19 structure (VGG19-TL-RF), transfer learning with Gaussian kernel SVM based on VGG-19 structure (VGG19-TL-SVM), VGG-19 and AlexNet. The 5-fold cross validation scheme validated deep learning models. When there was a slight change in the number of categories, retinal diseases were classified in accordance with its importance (judged by T.K.Y) as follows: BDR, PDR, dry AMD, wet AMD, RVO, RAO, Hypertensive retinopathy, Coat's disease, and retinitis. We adopted a grid search where ranges of parameter values were tested in order to obtain the optimal result from RF and SVM. We conducted the grid search in all dataset of 10 categories. A radial basis kernel function with a penalty parameter *C* of 100 and scaling factor *σ* of 10 determined the optimal model of SVM. 1,000 trees and five predictors for each node for RF were optimal. Since transfer learning approaches did not train the huge deep learning structure, the only a short time was needed to perform the grid search process in order to decide parameters. We did not perform left-right alignment of fundus images to identify the generalized performance of deep learning algorithms. We employed the NVIDIA GEFORCE GTX1060 3GB GPU for transfer learning and GTX980 6GB for SGD with Intel core i7 processor to train deep learning models more rapid.

## Results

**Fig 2:**
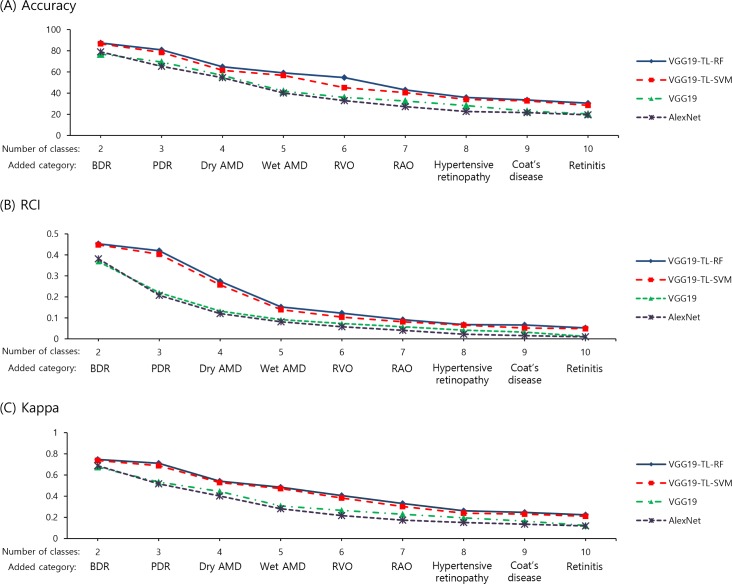
Performance of deep learning methods with 5-fold cross validation according to the number of categories. (A) the performance plot of accuracy (B) the performance plot of relative classifier information (C) the performance plot of Kappa. AMD, age-related macular degeneration; BDR, background diabetic retinopathy; PDR, proliferative diabetic retinopathy; RVO, retinal vein occlusion; RAO, retinal artery occlusion; VGG19-TL-RF, transfer learning with random forest based on VGG-19 structure; VGG19-TL-SVM, transfer learning with one-vs-one support vector machine based on VGG-19 structure.

Fig 2 shows results from this experiment with 5-fold cross validation for each number of categories. Two transfer learning methods (VGG19-TL-RF and VGG19-TL-SVM) exceeded the other two fully-trained deep learning models by using SGD (VGG-19 and AlexNet). VGG19-TL-RF worked well in all categories. The results varied depending on numbers of categories. As categories multiplied, performance of deep learning models underperformed. When only two categories (normal and BDR) were involved in the VGG19-TL-RF, the overall classification accuracy, RCI, and kappa were 87.4%, 0.453, and 0.747, respectively; whereas, the accuracy, RCI, and kappa were 30.5%, 0.052, and 0.224, respectively when all 10 categories were included.

**Fig 3:**
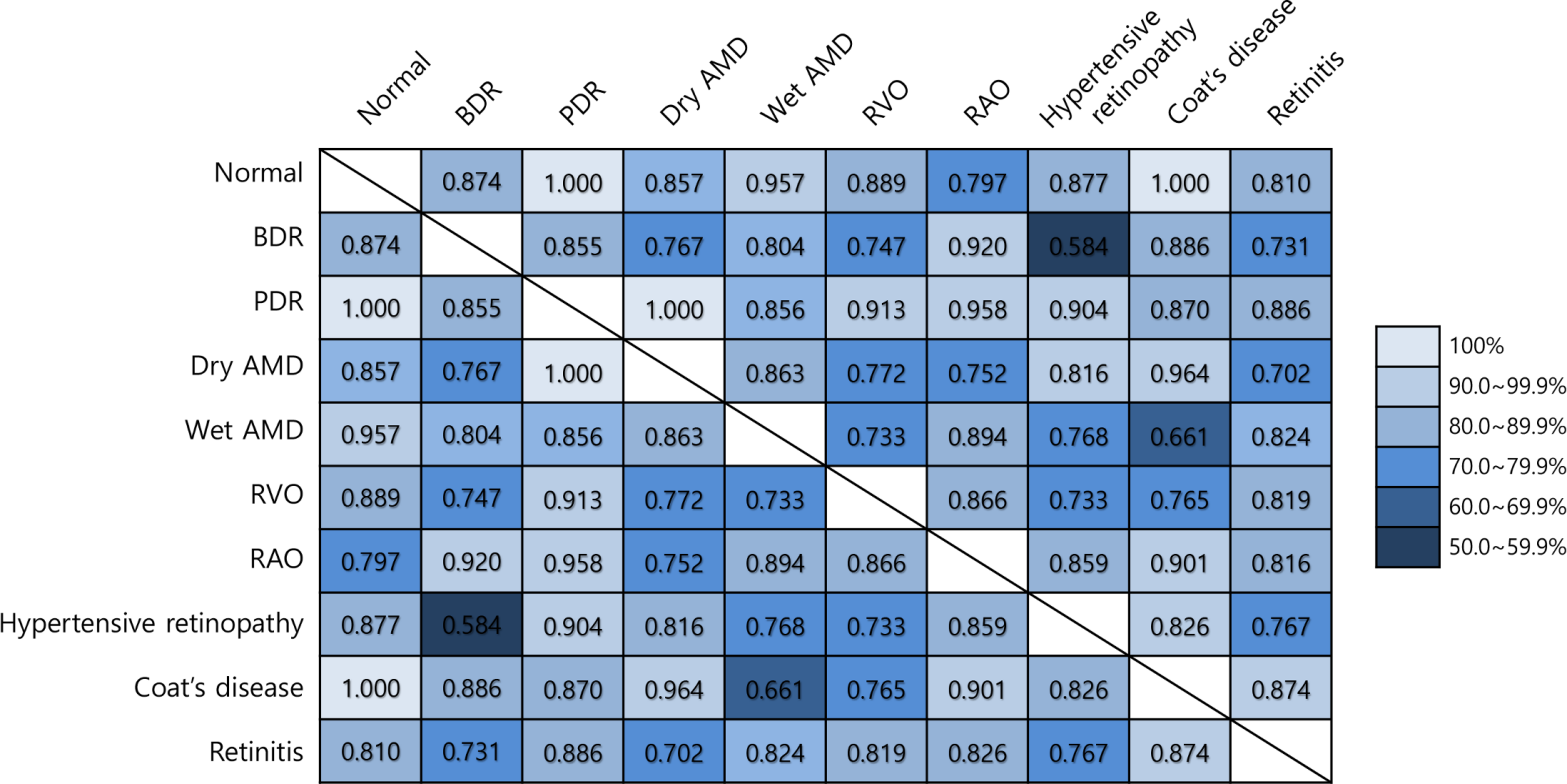
Binary discriminative accuracy between retinal diseases using transfer learning with random forest based on VGG-19 structure. The number of each pair shows the accuracy of binary classifiers.

We analyzed a detailed binary classification in order to determine categories that reduced the multi-categorical classification performance. We trained each binary classification model by using VGG19-TL-RF in all pairs of categories to examine binary discriminative powers between retinal diseases. Fig 3 presents an accuracy of 5-fold cross validation of pair-wise binary classification for all diseases. All diseases except RAO were separated from normal retina in which accuracy was over 80.0%. Discrimination between BDR and hypertensive retinopathy found the worst among all pairs (accuracy 58.4%). The accuracy of binary classifier discriminating wet AMD and Coat's disease showed 66.1%, which was lower than the mean accuracy.

**Fig 4:**
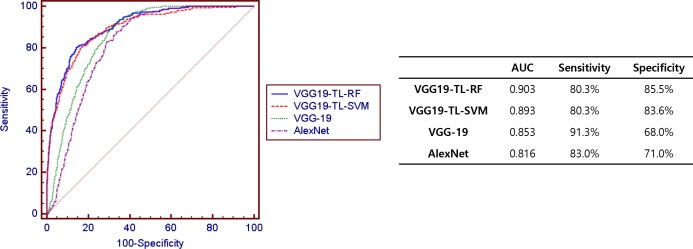
Receiver operating characteristic (ROC) curves of transfer learning with random forest based on VGG-19 structure (VGG19-TL-RF), transfer learning with random forest based on VGG-19 structure (VGG19-TL-SVM), and VGG-19, and AlexNet in predicting normal retina or retinal disease status using fundus photographs. We divided all data set (10,000 images) into training dataset (70%) and test dataset (30%). Retinal disease status includes diabetic retinopathy, age-related macular degeneration, retinal vein occlusion, retinal artery occlusion, hypertensive retinopathy, Coat’s disease, and retinitis.

We developed more general screening classification models to detect retinal abnormality. Fig 4 shows the ROC curves of each deep learning model for binary classification between normal or any disease status (normal versus abnormal). The VGG-TL-RF predicted abnormal retinal disease status (including 9 retinal diseases) with an area under the curve (AUC) of 0.903, sensitivity of 80.3%, and specificity of 85.5%. This result showed outperformance among others.

Considering the most important two disease groups including DMR and AMD, we combined images of normal, BDR, PDR, dry AMD, and wet AMD in the experiment as introduced in Table 1. VGG19-TL-RF also succeeded in all situations. For screening for early cases without progressed stages (a scenario of early disease screening), accuracy of VGG19-TL-RF using normal, BDR, and dry AMD was 72.8%, RCI of 0.283, and kappa of 0.577. When all disease categories on DMR and AMD (five categories: normal, BDR, PDR, dry AMD, and wet AMD) were included for more clinical situation, accuracy of VGG19-TL-RF was 59.1%, RCI of 0.151, and kappa of 0.485. When only DMR groups (normal, BDR, and PDR) were involved (a scenario of DMR staging), accuracy of VGG19-TL-RF found 80.8%, RCI of 0.420, and kappa of 0.711. When AMD groups (normal, dry AMD, and wet AMD) were included (a scenario of AMD staging), accuracy of VGG19-TL-RF was 77.2%, RCI of 0.371, and kappa of 0.657.

**Table 1:** Results from multi-categorical deep learning models for different approaches combining fundus images of normal, diabetic retinopathy and age-related macular degeneration.

	Accuracy (%)	RCI	Kappa
Screening early diseases: Normal + BDR + dry AMD (3 categories)			
VGG19-TL-RF	72.8	0.283	0.577
VGG19-TL-SVM	70.3	0.268	0.562
VGG-19	62.0	0.199	0.485
AlexNet	60.2	0.174	0.459
Normal + BDR + PDR + dry AMD + wet AMD (5 categories)			
VGG19-TL-RF	59.1	0.151	0.485
VGG19-TL-SVM	56.7	0.139	0.472
VGG-19	41.9	0.091	0.308
AlexNet	40.1	0.081	0.282
DMR severity classification: Normal + BDR + PDR (3 categories)			
VGG19-TL-RF	80.8	0.420	0.711
VGG19-TL-SVM	78.4	0.403	0.688
VGG-19	69.3	0.220	0.533
AlexNet	65.3	0.207	0.517
AMD severity classification: Normal + dry AMD + wet AMD (3 categories)			
VGG19-TL-RF	77.2	0.371	0.657
VGG19-TL-SVM	76.2	0.365	0.642
VGG-19	65.9	0.201	0.488
AlexNet	65.0	0.192	0.475

AMD, age-related macular degeneration; BDR, background diabetic retinopathy; PDR, proliferative diabetic retinopathy; VGG19-TL-RF, transfer learning with random forest based on VGG-19 structure; VGG19-TL-SVM, transfer learning with one-vs-one support vector machine based on VGG-19 structure

**Fig 5:**
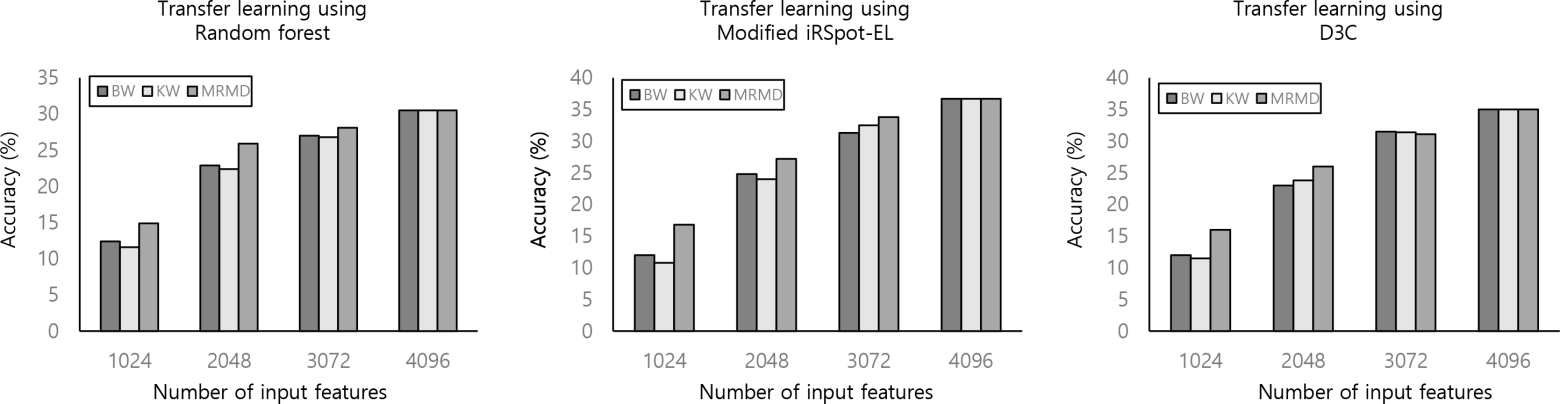
Comparison of different feature selection methods for 10 multi-categorical retinal image classification problem. KW, Kruskal-Walis one-way ANOVA; BW, ratio of features between-categories to within-category sum of squares; MRMD, Max-Relevance-Max-Distance.

We also analyzed the performance of ensemble learning in order to improve the deep learning classifier. Modified iRSpot-EL, multiple kernel learning, D3C, and deep SVM improved the classification performance in transfer learning setting (Table 2). In particular, the modified iRSpot-EL model (accuracy of 36.7%; RCI of 0.053; kappa of 0.225) performed better than other methods. When we reduced numbers of input features, MRMD feature selection methods raised the accuracy (Fig 5). The remaining algorithms (KW and BW) revealed a poor performance. Although feature selection was performed, transfer learning classifiers by using all input features achieved optimal performance.

**Table 2:** Performance results by using classic machine learning and ensemble classification for multi-categorical 10 retinal diseases classification problem in the VGG-19 transfer learning setting.

	Accuracy (%)	RCI	Kappa
Random forest	30.5	0.052	0.224
Support vector machine	28.5	0.048	0.210
Artificial neural network (2 hidden layers)	20.4	0.012	0.125
Modified iRSpot-EL (clustering approach)	36.7	0.053	0.225
D3C (K-means clustering with dynamic selection)	35.0	0.052	0.224
Multiple kernel learning	32.8	0.045	0.208
Deep SVM (AdaBoost)	35.2	0.051	0.223

## Discussion

This study is an investigation on multi-categorical deep learning algorithms for automated detection of multiple retinal diseases. Findings from this study revealed that the current deep learning algorithms were ineffective to classify multi-class retinal images from small datasets. It did not show effective and practical outcomes for a computer-aided clinical application. However, this paper suggests that the automated classifier should aim to discriminate at least normal, DMR, and AMD with multi-categorical classification due to importance of each disease in order to construct an automated retinal disease classification model by using deep learning model for the general public screening (especially for elderly).

The performance of deep learning models was dropped, as categories multiplied. In fact, this result is quite natural. When facing two categories (random performance: 50%), it is fairly common to have better performance than three-category situation (random performance: 33.3%). As categories multiplied, the expected accuracy at random distribution dropped. This result corresponded with previous studies [42]. A recent study that applied the GoogLeNet architecture for classifying skin cancer demonstrated that the growing number of classes proved underperformed (with an accuracy of 72.1% in a 3-class problem and 55.4% in a 9-class problem) [43]. An elaborate research should be conducted to augment this predictive power for multi-categorical classification. General clinicians observe that fundus image analysis on multi-categorical retinal disease diagnosis is difficult. For those unfamiliar with ophthalmology, they may find it more difficult than a binary classification problem on “one disease versus normal”. Although we used the new deep learning technique, diagnostic performance was inadequate to apply in clinical practice (S1 File–example of misclassification). Since every single disease has its own pathophysiological characteristics and displays retinal image under different patterns of progression, designing only one machine technique classifying multiple retinal diseases appears challenging. For example, a recent research showed that a specific method adopting Radon transform and discrete wavelet transform can detect AMD with an accuracy of 100% [44]. Therefore, it is necessary to construct disease-specific algorithms marking a distinction between retinal diseases in order to upgrade the performance of multi-categorical classification.

Recent medical images including fundus photograph were obtained with increased resolution. Our final goal is to build up a machine learning model to classify multi-category retinal diseases. The CNN method can provide high-level feature extraction and multi-category classification solution by using a huge and intensive computation system from high-resolution image. Although previous machine learning techniques reached high and successful achievements, they were difficult to generalize due to the absence of high-level abstraction [45].

However, there are also still several challenges to apply deep learning to clinical practice. One previous paper addressed ethical and political issues in terms of establishing database [46]. Collecting large-sized multiple retinal diseases data has been difficult for this reason. Another obstacle is that actual clinical issues consist of multiclass classification problems. Previous research concentrated on binary classification for retinal disease prediction. Although Google developed the deep learning model that works better than ophthalmologists, their model 'Inception-v3' based on the GoogLeNet structure was optimized to binary classification for DMR identification [17]. This model was trained by adding large image database collected only for DMR screening from diabetes patients. In fact, there are a couple of binary classification methods that showed similar performance in Google's deep learning model. A majority of previous studies with higher accuracy for automated DMR detection have been based on SVM [47,48]. RF can be used to assess automated DMR evaluation [49]. Abramoff's research group applied deep learning CNN to detect DMR and reported significantly improved performance [16].

This study suggests that further studies on automated diagnosis by using retinal image should identify multi-categorical classification. Binary classification models for DMR patients and healthy subjects were restricted under the clinical circumstance due to the prevalence of the several diseases. This is an obvious issue, yet researchers ignored this. According to previous epidemiologic studies, the prevalence rate of DMR and AMD in U.S. were 3.4% and 6.5%, respectively [50,51]. RVO can in no way be negligible either since the prevalence of RVO is reported to be about 0.5% [52]. Facing unlearned disease is more likely to occur for binary classification models. Therefore, there are problems in binary classification models for DMR in terms of the application for the clinical practice. One study suggested that researchers should consider screening for intermediate AMD and DMR simultaneously [5]. Binary classifiers cannot be applied into this approach because of results. However, there were a few studies on the development of a multi-categorical classification model in the field of ophthalmology. Most of these studies focused on the grading severity of diseases. Previously published papers claim that a concept of multi-categorical classification was applied to predict AMD progression by using SVM and RF [53]. Multiclass SVM also worked well in classification of DMR severity [54]. The latest research practiced deep learning CNN technique into a 5-class grading of DMR [55]. This study used C4.5 and a random tree method for multi-categorical classification of DMR and glaucoma [56]. However, this study misunderstood glaucoma, because glaucoma is not a retinal disease and cannot be diagnosed by only utilizing fundus photos [57]. To the best of our knowledge, there has been no research that identified multiple retinal disease groups by utilizing machine learning techniques.

Deep transfer learning model with RF performed better than other well-trained models. We used pre-trained CNN (trained using the ImageNet) in order to add data into a new feature space by spreading the fundus image database to the CNN due to lack of fundus image database available for training deep learning models. We applied traditional multi-categorical machine learning technique including RF and SVM for transfer learning. Although fundus photograph data was actually increased, fully-trained models (VGG19 and AlexNet) failed to exceed a small number of training images. Previous research revealed that transfer learning showed excellent performance comparable to fully-trained deep learning (trained with scratch) [24,58]. Although they are untrained and used fundus images, our results also revealed that transfer learning might be appropriate for multi-categorical classification of fundus images since pre-trained models included a wide range of powerful pattern extractors such as color, texture, and shape. RF was identified as the most robust machine learning classifier in previous researches [26,59]. Although classifiers were used in transfer learning, findings were consistent with previous findings. RF do not trigger problems due to over-fitting; whereas, the SVM undergoes a fine tuning process in order to avoid over-fitting and further reveal a high performance [26].

The current study has several limitations. First, a small number of images from a single study database produced fundamental limits. Many deep learning researchers come to agreement that such a small number of each category is insufficient to test the effectiveness of the proposed method. Deep learning technique generally requires more than a million samples to train without overfitting [17]. We used data augmentation and transfer learning in order to overcome this challenge. Nevertheless, the finding from performance proved to be unfulfilled. A series of trials and errors identified in this study will develop adequate methods for further studies. In addition, there was no external validation dataset to confirm the performance of classification models. If additional studies are performed, researchers should consider more retinal images from multicenter by including multiple ethnicities for a concrete training process. Second, our dataset has limited categories of retinal disease. Although we confirmed high prevalence of major retinal diseases, we missed several important retinal diseases such as retinal detachment, chorioretinal melanoma, and myopic degeneration. A multicenter scale project is needed for gathering more detailed fundus image data on rare and crucial diseases. Third, the reference diagnosis of the STARE database was often ambivalent despite comments by an ophthalmologist on all fundus images. Accurate diagnosis of retinal diseases should be verified by optical coherence tomography or fluorescein angiography [60]. However, the STARE database did not provide more detailed diagnosis. Further research should include retinal images diagnosed by clinical standard methods.

In this paper, we investigated multi-categorical classification of deep learning for automated diagnosis by using fundus photograph. Prediction models in this pilot study failed to show the advantage of using the deep learning employing multi-class retinal image datasets due to small size of datasets regarding classification performance. However, this is the primary attempt to construct deep learning models for multi-categorical classification problem of multiple retinal diseases. Importantly, diagnosis with a new deep learning technique underperformed as numbers of disease categories increased. Ensemble classifiers such as clustering and voting approach, dynamic selection, and AdaBoost could boost the classification performance for detecting multi-categorical retinal diseases. Further studies should focus on the construction of an extended prediction model with a diverse range of retinal diseases by applying multi-categorical classification techniques and sufficient amounts of datasets collected from hospitals. This study will provide proper ways to ophthalmologists who continue researching on deep learning in terms for clinical use.

## Supporting information

S1 File Example of misclassification. (PDF)
