# Supplementary material for: Multi-categorical deep learning neural network to classify retinal images: A pilot study employing small database
Source: PLoS One. 2017 Nov 2;12(11):e0187336. doi: 10.1371/journal.pone.0187336 (PMC5667846; doi:10.1371/journal.pone.0187336)
Supplement: S1 File — (PDF) [file pone.0187336.s001.pdf]

Multi-categorical deep learning neural network to classify retinal images: A pilot study employing small database

Running title: Multi-categorical Deep Learning with Retinal Images

Joon Yul Choi[1], Tae Keun Yoo[2]*, Jeong Gi Seo[2], Jiyong Kwak[2], Terry Taewoong Um[3], Tyler Hyungtaek Rim[2]*

[1]Department of Electrical and Computer Engineering, Seoul National University, Seoul, South Korea
[2]Institute of Vision Research, Department of Ophthalmology, Yonsei University College of Medicine, Seoul, South Korea
[3]Department of Electrical & Computer Engineering, University of Waterloo, Waterloo, Ontario, Canada

Supporting Information File S1. Example of misclassification.

	Ground truth	VGG19-TL-RF<br>Classification result
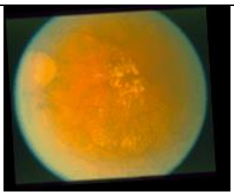	**Wet AMD**	**Dry AMD**
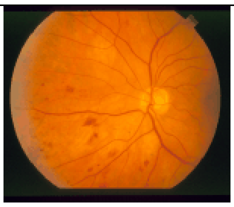	**RVO**	**BDR**
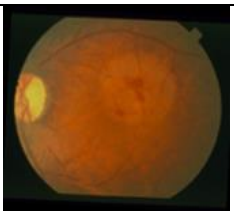	**Wet AMD**	**BDR**
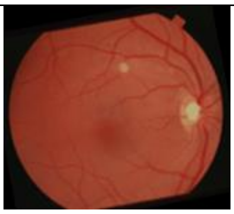	**BDR**	**Dry AMD**
